# Development of compounds for targeted degradation of mammalian cryptochrome proteins

**DOI:** 10.1098/rstb.2023.0342

**Published:** 2025-01-23

**Authors:** Jack Munns, Andrew D. Beale, Iacovos N. Michaelides, Sew Y. Peak-Chew, Andrei Mihut, Christine T. Major-Styles, Aiwei Zeng, R. Ian Storer, Rachel S. Edgar, Kevin Moreau, John S. O'Neill

**Affiliations:** ^1^ MRC Laboratory of Molecular Biology, Francis Crick Avenue, Cambridge CB2 0QH, UK; ^2^ Hit Discovery, Discovery Sciences, R&D, AstraZeneca, Cambridge CB2 0AA, UK; ^3^ Department of Infectious Disease, Imperial College London, London W2 1NY, UK; ^4^ Francis Crick Institute, 1 Midland Road, London, NW1 1AT, UK; ^5^ Safety Sciences, Clinical Pharmacology & Safety Sciences, R&D, AstraZeneca, Cambridge CB2 0AA, UK

**Keywords:** cryptochromes, circadian rhythms, cellular physiology, protein degradation, PROTAC, mass spectrometry

## Abstract

The mammalian cryptochrome proteins (CRY1 and CRY2) are transcriptional repressors most notable for their role in circadian transcriptional feedback. Not all circadian rhythms depend on CRY proteins, however, and the CRY proteins are promiscuous interactors that also regulate many other processes. In cells with chronic CRY deficiency, protein homeostasis is highly perturbed, with a basal increase in cellular stress and activation of key inflammatory signalling pathways. Here, we developed tools to delineate the specific effects of CRY reduction, rather than chronic deficiency, to better understand the direct functions of CRY proteins. Performing a bioluminescence screen and immunoblot validation, we identified compounds that resulted in CRY reduction. Using these compounds, we found that circadian PERIOD2 (PER2) protein rhythms persisted under CRY-depleted conditions. By quantitative mass spectrometry, we found that CRY-depleted cells partially phenocopied the proteomic dysregulation of CRY-deficient cells, but showed minimal circadian phenotypes. We did, however, also observe substantial off-target effects of these compounds on luciferase activity and could not ascertain a specific mechanism of action. This work therefore highlights both the utility and the challenges of targeted protein degradation and bioluminescence reporter approaches in disentangling the contribution of CRY proteins to circadian rhythmicity, homeostasis and innate immune regulation.

This article is part of the Theo Murphy meeting issue ‘Circadian rhythms in infection and immunity’.

## 1. Introduction

The mammalian cryptochrome proteins (CRY1 and CRY2) are widely interacting transcriptional repressors important for circadian rhythms and cellular homeostasis more broadly [1–10]. Related to ancient DNA repair photolyases, CRY protein homologues are evolutionarily widespread and required for normal daily rhythms in physiology across animal and plant kingdoms [11–13]. In mammals, CRY proteins are most notable as critical components in a well-characterized auto-regulatory transcription-translation feedback loop (TTFL), in which approximately 24 hour (circadian) oscillations in transcription are generated endogenously and cell autonomously [14,15]. In this system, CRY proteins act in complex with PERIOD (PER) proteins to bind to and inactivate their transcriptional activators (CLOCK/NPAS2 and BMAL1) [9,10,16]. In so doing, the CRYs and PERs rhythmically repress their own transcription and also facilitate daily rhythms in the abundance of many protein-coding and non-coding mRNA transcripts [17].

Within the cell-autonomous circadian timing system, the role of the CRY proteins is likely to be more complex than suggested above, however. The cycling expression of CRY proteins is not essential to their circadian function [18,19], and the peak nuclear abundance of CRY1 protein is much higher and occurs later than PER2 [20]. Early reports suggested that CRY-deficient cells and mice were entirely incompetent to sustain any form of circadian rhythm [21–25]. Subsequent reports, however, revealed various post-transcriptional and post-translational circadian rhythms that, whilst clearly impaired in comparison with wild-type controls, can persist in *Cry1*^−/−^; *Cry2*^−/−^ (CKO) cells, tissues and mice under some conditions. In CKO mouse suprachiasmatic nuclei (SCN) for example, one study found PER2::LUCIFERASE (PER2::LUC, translational reporter) rhythms to be detectable in ~40% of organotypic tissue slices from neonates but not adults [26,27]. Short periods and rather unstable rhythms of locomotor activity have also been observed in CKO mice under specific environmental conditions [28,29]. Transcriptional oscillations have not been detected in cultured CKO fibroblasts, but PER2::LUC translational rhythms have been reported in ~30% of recordings, albeit with greater variance (or reduced robustness) in amplitude and period length than wild type (WT) controls [28]. Circadian variations in protein abundance, phosphorylation and ion concentration have also been detected in cultured CKO fibroblasts [8]. As such, while important for physiological rhythms, the precise role(s) of CRY proteins in the context of daily biological timing remain to be fully elucidated.

Beyond circadian timing, the CRY proteins are promiscuous interactors and disrupted CRY function has wide-ranging consequences for cellular homeostasis. The CRY proteins widely associate with DNA, more so than other TTFL proteins [30], consistent with their interactions with a broad range of transcription factors. These include the cell-cycle-associated E2F-family transcription factors and numerous members of the nuclear receptor superfamily [3,31]. Concordantly, in two examples of the latter, deletion of CRY1 and CRY2 reportedly resulted in abnormal expression of target genes: PPARδ [32], which regulates lipid metabolism [33] and the glucocorticoid receptor [4], the pleiotropic regulator of glucose homeostasis, immunosuppression and other diverse cellular processes [34,35]. As well as disrupted gene expression, CKO fibroblast cells have been found to be substantially altered in terms of proteome composition. More than 33% of detected proteins are differentially abundant in CKO compared with WT fibroblasts, accompanied by reduced proteasome activity, increased translation, increased integrated stress response and increased sensitivity to proteotoxic stress [8]. In addition, the CRY proteins and their immediate interactors have been shown to influence the immune system. For example, CKO fibroblasts exhibit constitutive proinflammatory nuclear factor-kappa B (NF-κB) signalling and CKO mice exhibit autoimmunity symptoms [5,7]. Conversely, small molecule stabilization or overexpression of CRY proteins has been shown to suppress inflammation and inflammatory cytokines [5,6,36]. Through their numerous direct and indirect interactions, therefore, CRY protein activity is important for cellular homeostasis and immunity.

**Figure 1.**
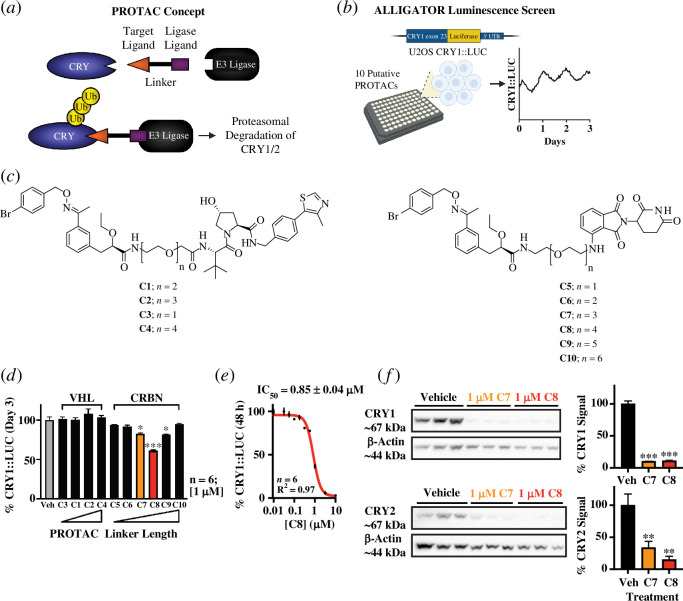
Development of compounds that result in effective depletion of CRY1 and CRY2. (*a*) PROTAC concept: a small molecule CRY ligand is conjugated with a ligand for the E3 ligase, CRBN or VHL, via a PEG linker. Upon binding by an effective PROTAC, CRY would be ubiquitinated by CRBN and consequently targeted to the 26S proteasome for degradation. (*b*) Screening approach: putative compounds were screened using a U2OS reporter cell line expressing a CRY1::LUC fusion protein, generated by knocking firefly luciferase into the endogenous CRY1 locus, followed by bioluminescence imaging in live cells. (*c*) Chemical structures of the **KS15**-comprising PROTACs designed to harness the VHL (left) and CRBN (right) E3 ligases. (*d*) Percentage of vehicle CRY1::LUC bioluminescence (in relative light units, RLU) following treatment with respective putative CRY degrader compounds, **C1−10**, at 1 μM. E3 ligase ligand used and relative length of PEG linker are indicated. Bars show mean ± s.e.m. of six replicates, and show the median luminescence value of each replicate on the third day (48–72 h) following compound addition, expressed as a percentage of the vehicle (0.01% DMSO) mean. Statistics: Kruskal Wallis with Dunn’s multiple comparisons test (**C7**: *p* = 0.0167; **C8**: *p* = 0.0006; **C9**: *p* = 0.0104); *n* = 6 replicate populations of cells. (*e*) Dose–response for dilution series after addition of **C8**. Data are presented as percentage of mean vehicle (0.1% DMSO) CRY1::LUC bioluminescence signal (RLU) after 48 h treatment. Points show mean ± s.e.m.; *n* = 6 replicate wells. Solid red line indicates a variable sigmoidal fit; with associated IC_50_ and *R*^2^ values also reported. (*f*) Immunoblots for CRY1 and CRY2, respectively, following 60 h incubation with vehicle (0.01% DMSO), compound **C7** or **C8**, normalized to β-actin. Bars show percentage of mean vehicle intensity (mean ± s.e.m.). Statistics: one-way ANOVA with Holm–Šídák multiple comparisons test (CRY1 adjusted *p*-values: **C7**: *p* = 0.0007; **C8**: *p* = 0.0007; CRY2: **C7**: *p* = 0.008; **C8**: *p* = 0.0049); *n* = 3 independently treated populations of U2OS PER2::LUC cells.

Studies of CRY protein function have hitherto been limited to highly perturbed knockout models that lack CRY protein function entirely, complementation assays [10,19] and small molecule modulators that stabilize CRYs or inhibit select interactions [37–43]. Here, we developed tools to deplete CRYs using a putative proteolysis targeting chimaera (PROTAC) design. PROTACs are a well-established modality for targeted protein degradation, including some compounds in phase I and II clinical trials for therapeutic use [44,45]. They are heterobifunctional molecules comprising a ligand for the target protein, conjugated with an E3 ligase ligand via a linker. Upon binding the target and E3 ligase proteins, PROTACs induce E3-mediated ubiquitination and subsequent proteasomal degradation of the target protein [44,45] (figure 1*a*). By applying this novel approach to proteins involved in circadian timekeeping, we directly assess CRY contribution to cellular rhythms and proteome integrity. We also describe the challenges encountered when screening these compounds, with respect to bioluminescence assays, off-target effects and mechanism of action experiments that suggest our compounds do not act via a canonical PROTAC mechanism of action.

## 2. Results

### (a) Development and screening of putative CRY-degraders

To achieve acute depletion of CRY proteins, we designed putative PROTAC compounds. A 2-ethoxypropanoic acid derivative, **KS15**, was previously shown to enhance E-box-mediated transcription [37,41,42]. The authors subsequently synthesized an amide-linked biotinylated probe, by exploiting **KS15**’s carboxylic acid group, and used it to pull down CRY1 and CRY2 from HEK293T cell lysates [41]. This experiment demonstrated that the carboxylic acid group’s functionality is not essential for binding the CRYs and that an amide linkage is tolerated. By similarly modifying **KS15**, 10 putative PROTACs carrying an analogous amide linker were designed with the aim of acutely depleting CRY proteins. **KS15** was conjugated via polyethylene glycol (PEG) linkers of varying length to a ligand for one of two E3 ligases: VH032 for Von Hippel-Lindau (VHL) or the immunomodulatory drug (IMiD), pomalidomide, for cereblon (CRBN) (figure 1*c*).

To screen these compounds for effective CRY protein degradation, we employed an *in vitro* bioluminescence reporter approach. We genome-edited the human U2OS cell line to express firefly luciferase fused in-frame with the C-terminal exon of endogenous CRY1 (electronic supplementary material, figure S1a). This enabled proxy measurement of endogenous CRY1 protein levels over time via bioluminescence monitoring using an ALLIGATOR system [46] (figure 1*b*). We found that a 1 μM treatment with several of our putative CRBN-recruiting compounds significantly reduced CRY1::LUC signal, while putative VHL-recruiting compounds had no significant effect on luminescence following a 3 day treatment (figure 1*d*). Subsequent removal of the compounds mostly rescued these effects 24–48 h later (electronic supplementary material, figure S1c). Consistent with prior observations [47,48], we observed that putative CRBN-recruiter efficacy increased with PEG linker length to an optimum, and decreased at greater lengths (figure 1*c*,*d*). Compound **C8**, with an intermediate length linker (*n* = 4 repeating units), led to the greatest reduction in CRY1::LUC signal (figure 1*d*), with an IC_50_ of ~1 μM (figure 1*e*). This compound led to no significant effect on cell viability after 72h treatment with concentrations of 1 μM and below (albeit with a very modest reduction in the number of cells; electronic supplementary material, figure S1d). Some cell death was observed at concentrations >3 μM and over longer treatment durations (data not shown). A 1 μM treatment was therefore used henceforth.

We confirmed the depletion of both CRY1 protein and its paralogue, CRY2, by immunoblot assay. Cells were treated with either **C8**, or **C7**, a compound with a shorter linker length that elicited a significant but more modest reduction in CRY1::LUC activity (figure 1*d*,*f*; electronic supplementary material, figure S1*e*). Informed by the luminescence data (figure 1*d*), compounds were applied over a period of 60 h (a duration of treatment for which differences in CRY1::LUC activity could be effectively discerned; figure 1*d*). In this assay, however, both compounds reduced cellular CRY levels to just above the limit of detection. This more marked reduction in protein abundance relative to that observed using indirect CRY1::LUC measurements is likely explained by luminescence correlating more closely with the level of nascent synthesized fusion protein than total CRY1::LUC abundance; catalytic inactivation of the luciferase enzyme occurs within 1–2 h [49], while CRY1 (and the luciferase protein itself) have longer half-lives [50,51]. Consequently, live cell luciferase activity measurements would be expected to underestimate the actual decrease in CRY protein levels.

### (b) CRY degrader specificity and effects on circadian rhythms

**Figure 2.**
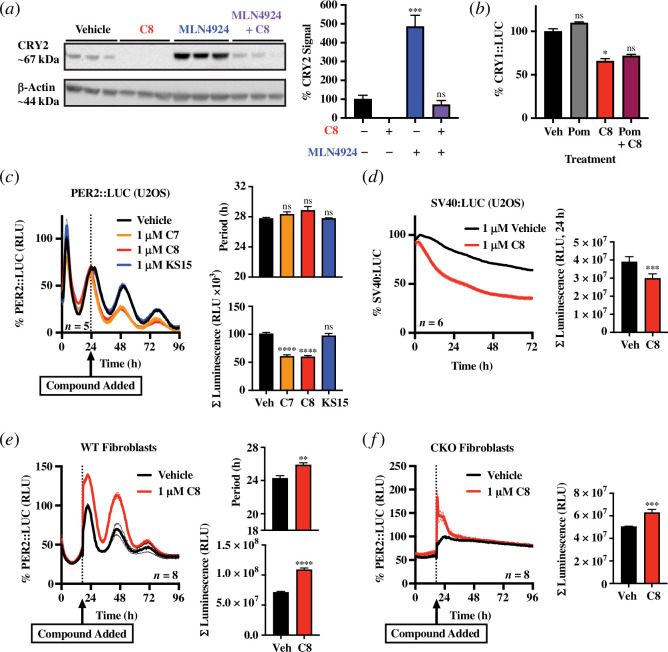
Effective CRY degraders have on- and off-target effects and do not ablate circadian rhythms in bioluminescence in human and mouse cells. (*a*) Immunoblot for CRY2 following 6 h pre-incubation with MLN4924 followed by 48 h incubation with 1 μM **C8** and respective controls, normalized to β-actin. Bars show percentage of vehicle (0.03% DMSO final) mean intensity ± s.e.m. Statistics: one-way ANOVA with Holm–Šídák multiple comparisons test (adjusted *p*-values: MLN4924: *p* = 0.0008; MLN4924+**C8**: *p* = 0.608); *n* = 3 independently treated populations of U2OS cells. (*b*) CRY1::LUC in cells treated with 1 μM **C8** following 24 h preincubation with 30 μM pomalidomide. Bars show mean ± s.e.m. of 3–5 replicates and show the median luminescence value of each replicate in the 24–48 h following **C8** addition expressed as a percentage of the vehicle (0.01% DMSO) mean. Statistics: Kruskal Wallis with Dunn’s multiple comparisons test; *n* = 3–5 populations of cells. (*c-e*) Longitudinal bioluminescence recordings and circadian parameters in luciferase reporter cell lines treated with putative CRY degraders and associated compounds. Period estimated by fitting a damped cosine wave to individual replicate data (see methods). Total luminescence is calculated as the sum of luminescence (in relative light units, RLU) over the first 24 h window following treatment. All bar graphs show mean ± s.e.m. Statistics: respective one-way ANOVAs with Holm–Šídák multiple comparisons tests (*p*-value threshold = 0.05, *p < 0.05; **p < 0.01; ***p < 0.001; *p < 0.0001). (*c*) PER2::LUC luminescence in U2OS cells following treatment with 1 μM **C7**, **C8** or **KS15**. Luminescence presented as a percentage of vehicle (0.01% DMSO) mean at its pre-treatment peak. (*d*) Constitutive SV40:LUC luminescence in U2OS cells following treatment with 1 μM **C8** (treatment applied at time 0). Luminescence presented as a percentage of vehicle (0.02% DMSO) mean at its post-treatment peak. (*e, f*) PER2::LUC luminescence in WT (*e*) and CKO (*f*) mouse lung fibroblasts before and following treatment with 1 μM **C8**. Luminescence presented as a percentage of vehicle (0.03% DMSO) means at respective post-treatment peaks.

We next sought to determine whether CRY protein depletion by **C8**, the most effective compound in bioluminescence assays, was E3 ligase-dependent as expected for a canonical PROTAC mechanism of action. We incubated cells with 1 μM MLN4924, which prevents ubiquitination by cullin-RING E3 ligases such as CRBN by inhibiting Nedd8-activating enzyme [52]. Cells were preincubated for 6 h prior to the addition of **C8**, and lysed at a comparable timepoint to those in figure 1*f*. Immunoblot analysis revealed MLN4924 treatment was sufficient to rescue CRY2 protein abundance in **C8**-treated cells to levels comparable to the vehicle treatment (figure 2*a*). However, E3 ligase inhibition alone significantly increased CRY2 levels relative to vehicle and rescue of CRY2 by MLN4924 was incomplete relative to this condition. This could arise either because 1 μM MLN4924 did not achieve saturating inhibition of the ubiquitin-proteasome system (UPS), or because the CRY-depleting activity of **C8** is not entirely mediated by the UPS, or a combination of the two. Complementary to this, we performed ‘competition’ bioluminescence assays in which cells were pre-treated with **C8**’s E3 ligase ligand, pomalidomide, prior to **C8** addition and found the efficacy of **C8** CRY reduction was unaffected (figure 2*b*). Collectively these results suggest that while CRY is reduced by **C7** and **C8**, we cannot conclude that this occurs via a canonical PROTAC mechanism of action.

As we had identified compounds that reduce both CRY1 and CRY2 proteins in cells, we sought to test the effect of acute CRY depletion on the circadian system using real-time circadian bioluminescence reporters. We employed U2OS cells expressing the CRY binding partner, PER2, fused to luciferase at the endogenous locus (PER2::LUC, a well-established translational circadian clock reporter system [53,54]). Notably, circadian rhythms in PER2::LUC continued in the presence of both **C7** and **C8** compounds, showing that PER2 protein rhythms can persist largely unperturbed when both CRY proteins are acutely depleted (figure 2*c*). These data are consistent with prior observations of PER2::LUC rhythms in CRY1/2 double knockout (CKO) mouse fibroblasts, which can persist over multiple circadian cycles under constant conditions [28]. In contrast to the CRY-binding ligand alone, **KS15**, which should stabilize CRY1/2 [37,41,42], we noted significantly reduced amplitude and overall PER2::LUC luminescence in the presence of 1 μM **C7** and **C8**. This observation is superficially consistent with expectations from the canonical TTFL model, where interactions with CRY1/2 are thought to increase PER protein stability and therefore increase feedback repression at the PER promoters [14,15,55,56]. Critically, however, we also observed that **C8** reduced the activity of constitutively expressed luciferase (SV40:LUC) to a similar extent (figure 2*d*), which suggests **C8** has off-target effects that directly or indirectly affect the activity of the firefly luciferase reporter.

**KS15** has also been reported to disrupt the circadian system in mouse cells (via disrupted binding to BMAL1 [37,41,42], which is essential for circadian rhythmic transcriptional repression [9,10,16]). We therefore next extended our circadian reporter approach to mouse fibroblasts expressing the PER2::LUC translational reporter [54]. Circadian rhythms persisted upon **C8** application to PER2::LUC mouse adult fibroblasts, with significant but modest effects on the period of oscillation (figure 2*e*), similar to the nonsignificant effect seen in U2OS cells (figure 2*c*). In contrast to U2OS cells, the overall PER2::LUC signal was significantly higher in **C8**-treated cells relative to the vehicle. We also did not observe such changes in luminescence when treating with **KS15** alone (electronic supplementary material, figure S2c). However, a similar increase in overall luminescence was also observed for genetically CRY-deficient cells during the initial 24 h following **C8** treatment (figure 2*f*). It is therefore plausible that, as for U2OS cells, **C8** has off-target effects that directly or indirectly affect the activity of the firefly luciferase reporter.

Off-target effects of small molecule activators and inhibitors on luciferase activity are a well-known pitfall of cellular bioluminescence assays [57–59], but to our knowledge, this is the first time they have been observed for any putative PROTAC compound. To gain insight into the generality of such off-target effects, we applied four validated commercial PROTACs that target the transcriptional cofactor BRD4 to U2OS PER2::LUC cells (two VHL and two CRBN-recruiting degraders). As with **C7** and **C8**, two of the BRD4 PROTACs reduced overall luciferase activity (one VHL, one CRBN-recruiting; electronic supplementary material, figure S2*a,b*). Because all four BRD4 PROTACs have previously been shown to deplete BRD4 in human cells [60–63], but only two elicited acute reductions in luciferase activity and this reduction was not specific to a particular recruited ligase, we suggest that off-target effects of PROTAC compounds on luciferase activity may be relatively common.

### (c) Proteomic analysis of C8-treated and CRY knockout cells

Irrespective of mechanism-of-action or off-target effects, our immunoblot analyses revealed that incubation with compound **C8** for >48 h evidently reduces CRY1 and CRY2 protein abundance (figures 1*e,f* and 2; electronic supplementary material, figure S1e). Consistent with this, using a TMT-quantitative mass spectrometry approach, we found that treatment of U2OS cells with **C8** elicited a profound upregulation of many transcription factors (in accordance with the role of the CRY proteins as transcriptional repressors). Ranked Gene Ontology (GO) analysis using the online tool GOrilla [64–67] revealed an overwhelming enrichment of GO transcriptional function terms among the upregulated proteins (electronic supplementary material, figure S3a). Associated with the GO term with the lowest *p*‐value (GO:0000978, relating to promoter binding by RNA polymerase II), were numerous transcription factors that have been shown to be CRY-regulated or associated, including NFIL3 [68], REV-ERBβ [69], CEBPB [70], BHLHE41 [71], SREBF1 [72] and p53 [73] (electronic supplementary material, figure S3*b*). Using this approach, we did detect CRY1 itself (but not CRY2), which was modestly reduced after a 24 h treatment with **C8** (electronic supplementary material, figure S3*c*).

Having developed a compound that does reduce cellular CRY abundance, we wanted to investigate whether our compound could distinguish acute effects of CRY depletion from chronic CRY deficiency. Specifically, we wanted to determine to what extent depleting CRY in WT cells recapitulates the large-scale proteome dysregulation seen in genetically CRY-deficient (CKO) cells. Using a mouse fibroblast model, we had previously speculated this dysregulation to be an indirect adaptive response to gene loss-of-function rather than direct cryptochrome-dependent regulation of thousands of cellular proteins [8]. We sought to test this using the new CRY degrader, performing 16-plex TMT-quantitative mass spectrometry on vehicle or **C8**-treated WT and CKO mouse lung fibroblasts (using two lines isolated in the prior work and performing MS3-level quantification for the first time using these lines). For this experiment, we aimed to reduce CRY as completely as possible, treating cells with 1 μM **C8** over a period of 10 days (replenishing culture medium containing 1 μM **C8** every 72 h).

**Figure 3.**
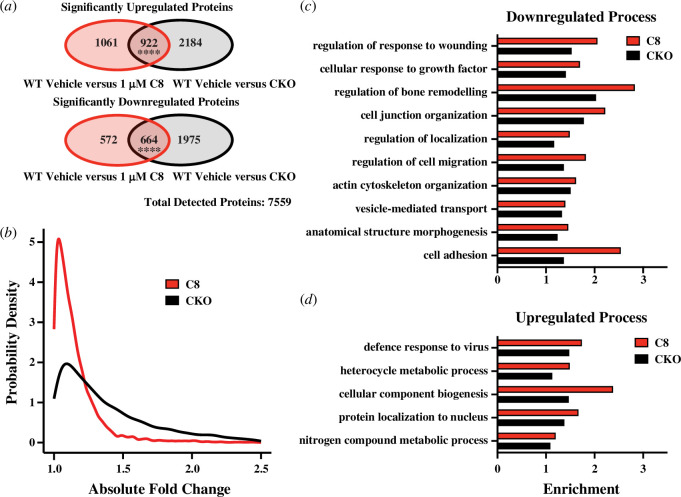
Mass spectrometry reveals CRY degraders replicate CRY knockout proteome dysfunction. (*a*) Numbers of proteins showing significant abundance increases or decreases in **C8**-treated WT and vehicle-treated (0.01% DMSO) CKO mouse fibroblasts, both with respect to vehicle-treated WT cells (two-way *t*‐test, BHQ < 0.05). Significance of overlapping proteins determined by Fisher’s exact tests (four asterisks, BHQ value < 0.0001). (*b*) Probability density plot showing absolute-value fold change in all 7559 proteins in **C8**-treated WT cells and CKO cells, both with respect to vehicle-treated WT cells (full list of proteins in electronic supplementary material, table S6). Distributions were compared using a Kolmogorov–Smirnov test; *p*‐value < 0.0001. (*c,d*) Bar charts showing selected enriched GO Process terms associated with individual lists of significantly downregulated (*c*) and upregulated (*d*) proteins from (*a*). Full lists of terms given in electronic supplementary material, tables S1,2. Shown are terms that co-occurred in **C8**-treated WT and vehicle-treated CKO ontology lists (*p*-value threshold 0.01). Omitted are terms enriched **C8**-treated CKO relative to vehicle-treated CKO.

Consistent with previous observations [8], the CKO proteome was considerably different to WT in terms of protein abundances; of 7559 detected proteins, 5745 (76%) significantly differed relative to the WT vehicle condition (*t*-tests with Benjamini–Hochberg-corrected *q*, BHQ < 0.05; figure 3*a*). In **C8**-treated WT cells, we observed abundance changes in a substantial, but smaller proportion of the proteome (3219 proteins, 43%; figure 3*a*). Correspondingly, in terms of effect size, the absolute fold change value for all 7559 proteins was generally much greater for cells where CRY had never been present than when it was depleted over 10 days with **C8** (figure 3*a*). Comparing the identities of altered proteins, many of the proteins in **C8**-treated WT cells also differed in vehicle-treated CKO cells. Of these, a greater proportion than expected by chance moved in the same direction (being upregulated or downregulated respectively in both **C8**-treated and CKO cells). Collectively, these results thus suggest a partial, but incomplete phenocopying of the CRY-deficient cellular environment in CKO cells was achieved by **C8** treatment (figure 3*a*).

To investigate the specific cellular processes and functions impacted by CRY reduction, and compare these to chronic CKO deficiency, we performed GO analysis using GOrilla [64–67]. We specifically looked for convergent terms between significantly upregulated and significantly downregulated proteins in **C8**-treated WT cells and vehicle-treated CKO cells. Comparing to a background list of all 7559 detected proteins, we identified a plurality of diverse ontology terms up- and downregulated in both **C8**-treated and CKO cells that were, in addition, not identified in **C8**-treated CKO cells (figure 3*c*,*d*; electronic supplementary material, S3*d*,*e* and tables S1–S4). The diversity of these terms is perhaps unsurprising, given that CRY proteins are broad interactors [3,4,30–32], but only some were consistent with known functions of CRYs. For example, protein localization (‘protein localization to nucleus’ and ‘regulation of localization’) is consistent with the described circadian role of CRY proteins in the nuclear translocation of PER [14,74,75]. Terms relating to the putative immune functions of CRY were also identified, but specifically included defence responses to viruses and wound healing functions, rather than terms relating to the previously reported anti-inflammatory role of CRYs [5–7,42]. Our observation that so many different classes of biological processes were similarly up- and downregulated when comparing CRY-deficient with CRY-depleted cells provides further validation of our CRY degradation approach.

## 3. Discussion

Our work aimed to expand on previous insights from small molecule inhibitors [37–43] and knockout models [2,8,28,32] by developing a small molecule to delineate the direct effects of CRY depletion from genetic deficiency in mammalian cells. To this end, using a novel CRY1::LUC reporter as a proxy for protein abundance allowed us to efficiently screen putative CRY degraders, and differentiate between VHL-recruiting (ineffective) and CRBN-recruiting (effective) compounds (figure 1*b*,*d*). Effective compounds were subsequently validated for CRY1 and CRY2 reduction efficacy by immunoblotting assays (figure 1*f*). We then used these compounds to investigate the effects of acute CRY reduction on circadian rhythms in PER2 translation (figure 2*c*,*e*) and proteome alterations in comparison with CKO cells (figure 3). On the other hand, we observed notable off-target effects. These included incomplete rescue of CRY levels in cells where CRBN activity was inhibited via MLN4924 (potentially indicating a mechanism of action deviating from a canonical PROTAC; figure 2*a*,*b*) and bioluminescence changes in constitutive SV40:LUC reporter and CKO cells (figure 2*d*,*f*). Our work therefore also highlights the challenges and potential pitfalls of bioluminescence reporter assays and developing targeted degraders against circadian proteins.

Our primary observation from PER2::LUC circadian bioluminescence assays was that rhythms persist in cells where CRY protein abundance was acutely reduced well below the habitual circadian nadir (figure 2*c*,*e*) [20]. The mammalian cryptochromes’ most well-studied circadian function is in interacting with PER proteins, facilitating PER nuclear entry and retention [14,74,75] and enabling circadian transcriptional repression [9,16]. CRY1 and CRY2 are partially redundant paralogues [24,25], but in the absence of both, rhythms in cellular transcription and mouse behaviour are reported to be shorter or damped beyond detection [21–23,25,28,29,76]. It is notable, therefore, that we observed no loss of rhythm or period shortening in bioluminescence assays. In mouse CKO SCN tissue, however, where PER2 is principally cytoplasmic, low levels of virally reintroduced CRY and subsequent PER2 translocation are sufficient to restore normal circadian rhythmicity [74]. This suggests that cells only require a baseline level of CRY protein expression for its circadian molecular function to be performed. Nuclear CRY1 proteins are also reported to be more abundant than PER2, and while oscillatory, consistently present [20]. Our results, in which PER2::LUC rhythms in **C8**-treated cells reflect substantial rather than total CRY depletion, are consistent with this latter work.

Our bioluminescence experiments also revealed apparent non-specific effects, including an acute induction of PER2::LUC in CKO fibroblasts upon treatment with the putative CRY degraders and a progressive reduction in constitutive U2OS SV40:LUC activity (figure 2*d*,*f*). We also observed a non-ligase or target-specific signal reduction in U2OS PER2::LUC using commercial BRD4 PROTACs (electronic supplementary material, figure S2*a,b*). The off-target effects we observed using different compounds could have arisen through undetermined interactions with endogenous macromolecules, or the luciferase reporter itself. Luminescence reporters have been used as a proxy measure of protein abundance in previous PROTAC development [77,78] and are one of the most common research tools within the circadian field [54,79]. However, diverse compounds have been shown to inhibit or stabilize luciferase [57–59,80], an enzyme with extensive sequence and functional homology to long-chain fatty acyl-CoA ligases (ACSL), which can rescue beta oxidation in ACSL-deficient cells [81]. We speculate that off-target effects on luciferase activity may occur because certain synthetic compounds contain specific combinations of functional groups and PEG linkers that interfere with luciferase via its ancestral CoA–ligase activity. The presence of **C8** off-target effects means we cannot conclusively ascribe some bioluminescence observations (the modest effects on circadian period for example; figure 2*c*,*e*) to CRY depletion. Our results highlight the need for caution and independent validation when interpreting luminescence data, particularly with respect to circadian biology.

Our putative CRY degraders enabled us to compare CRY-depleted WT cells with CRY-deficient knockout fibroblasts using quantitative mass spectrometry (figure 3). Consistent with previous work [8], we found most of the proteome (76%) to be significantly altered between WT and CKO (vehicle-treated) cells. Treatment with compound **C8** for 10 days also substantially changed the proteome of WT cells, but did not completely phenocopy the cryptochrome knockout cells, in terms of the number or extent of significantly changed proteins (43%) (figure 3*a*,*b*). This indicates non-equivalence of CRY reduction and genetic deficiency. CKO cells are disrupted at the level of the transcriptome, proteome and phosphoproteome, and show increased stress and susceptibility to stress [2,4,8], while CKO mice show considerable metabolic changes and health defects [2,82,83]. This is frequently interpreted to support a direct regulatory role for CRY in many different biological processes, whereas our work suggests some elements of the CKO phenotype may be indirect adaptions to the chronic absence of CRY protein function. We do however note that our 10 day treatment may itself include some indirect effects on protein homeostasis. Our work emphasizes the need to distinguish direct cryptochrome targets and effectors from potentially indirect consequences resulting from chronically dysregulated protein homeostasis in cells where CRY was never present.

With respect to the direct effects of CRY deficiency, GO analysis of our mass spectrometry data did reveal some similarities in functional enrichment between CRY degrader-treated WT and CKO (vehicle-treated) cells (figure 3*c*,*d*). For example, ‘regulation of response to wounding’, along with related terms (‘actin cytoskeleton organization’, ‘cell migration’ and ‘cell adhesion’) were specifically downregulated in both. Wound healing is a circadian process, more active in the day in humans, and has been shown to occur in mouse skin fibroblasts *in vitro*, with associated CRY-dependent rhythms in actin dynamics (F/G actin ratios) and cell adhesion [84,85]. Our work suggests that CRY protein deficiency reduces basal levels of the proteins that regulate this key fibroblast function, whether CRY is ablated genetically or pharmacologically. Similarly, our analysis uncovered a putative direct role for cryptochromes in antiviral defence, with basal levels of viral restriction factors upregulated in both WT **C8**-treated and CKO cells compared to WT vehicle-treated cells. Interestingly, a previous proteome comparison of WT and BMAL1-deficient cells showed no significant enrichment in either direction for pathways involved in antiviral defence [86], suggesting this may be a function of CRY proteins that is independent from the repression of BMAL1 transcription. Many viruses exhibit time-of-day variation in their replication, perhaps unsurprisingly given that most cellular systems co-opted by viruses exhibit circadian rhythmicity [87]. Whether cells exhibit CRY-dependent rhythms in antiviral protein levels and how this affects virus replication are open questions, addressed in an accompanying paper within this themed issue [88] .

Collectively, our work provides expanded insight into the functions of the cryptochrome proteins, while also highlighting the challenges inherent to bioluminescence reporter assays and tools that directly deplete circadian clock proteins. Targeted degradation via PROTACs has proven utility *in vitro* and in the clinic [44] and our study demonstrates the value of small molecules designed to deplete CRY1/2. With further optimization, such degradation approaches will further improve our molecular understanding of CRY and other circadian proteins, with potential uses including chronomodulatory drugs to treat circadian disorders [89].

## 4. Methods

### (a) Mammalian cell lines and maintenance

A U2OS cell line expressing the CRY1::LUCIFERASE fusion protein was generated by CRISPR-Cas9 homology-directed repair (HDR). Guide RNAs for Cas9 editing at the stop codon of *CRY1* were designed using CHOPCHOP v2 [90] with default parameters. Guide RNA sequences were inserted as annealed complementary single-stranded oligos into BbsI-digested pSpCas9−2A-GFP (PX458, Addgene). HDR templates were created by direct gene synthesis. Homology arms of ~1000 bp were included on either side of the insert coding sequence. In this way, human codon-optimized firefly *luciferase* is inserted immediately 5′ of the endogenous stop codon. U2OS cells were transfected with a 3:1 molar ratio of linearized HDR template: Cas9 sgRNA using PEI at 3 μg μg^−1^ DNA. On the third day after transfection, GFP-positive cells were single-cell sorted and expanded as clonal cell lines in 96-well plates. Clonal lines were screened for bioluminescence using an ALLIGATOR as detailed below. Positive clones were identified by the presence of oscillating luminescence brightness with a period of approx. 24 h in constant conditions. Positive clones were validated by PCR (electronic supplementary material, figure S1a).

PER2::LUC U2OS cells were generated as described in Beale *et al*. [53]. Wild-type and CRY1/2 double knockout (CKO) PER2::LUC mouse lung fibroblasts used here were previously isolated and immortalized in Wong *et al*. [8].

For general maintenance, cells were cultured at 37°C at 5% CO_2_ in Dulbecco’s modified Eagle’s medium (DMEM, high glucose, GlutaMAX^TM^, pyruvate; 31966-021, Gibco), supplemented with penicillin (100 units ml^−1^), streptomycin (100 µg ml^−1^; Gibco) and 10% HyClone serum (U2OS cells: FetalClone II, fibroblasts: FetalClone III; Cytiva) unless noted.

### (b) Compound synthesis and characterization

Putative PROTACs were synthesized from commercially available starting materials using standard amide-coupling protocols, as exemplified by the synthesis of (2*R*)−3-(3-((*E*)−1-(((4-bromobenzyl)oxy)imino)ethyl)phenyl)-*N*-(14-((2-(2,6-dioxopiperidin−3-yl)-1,3-dioxoisoindolin-4-yl)amino)-3,6,9,12-tetraoxatetradecyl)-2-ethoxypropanamide (**C8**) below (figure 1*c* and electronic supplementary material, S1*b*).

*(R*,*E)*-3-(3-(1-(((4-bromobenzyl)oxy)imino)ethyl)phenyl)−2-ethoxypropanoic acid (CAS: 1033781-20-2; 205 mg, 0.488 mmol) was added to a mixture of 4-((14-amino-3,6,9,12-tetraoxatetradecyl)amino)-2-(2,6-dioxopiperidin-3-yl)isoindoline-1,3-dione (CAS: 2225940-52-1; 160 mg, 0.325 mmol), hydroxybenzotriazole (HOBt, 99.0 mg, 0.646 mmol), 1-ethyl-3-(3-dimethylaminopropyl)carbodiimide (EDC; 125 mg, 0.652 mmol) and *N*,*N*-diisopropylethylamine (DIEA; 227 μl, 1.30 mmol) in dimethylformamide (DMF; 4 ml). The resulting mixture was stirred at room temperature for 16 h. The solvent was removed under reduced pressure and the crude product was purified by preparative HPLC (XBridge Prep OBD C18, 5 μm, 30 × 150 mm) using decreasingly polar mixtures of water (10 mmol l^−1^ NH_4_HCO_3_ + 0.05% NH_3_.H_2_O) and MeCN (elution gradient 55–70%). Fractions containing the desired compound were evaporated to dryness to afford (2*R*)−3-(3-((*E*)−1-(((4-bromobenzyl)oxy)imino)ethyl)phenyl)-*N*-(14-((2-(2,6-dioxopiperidin−3-yl)-1,3-dioxoisoindolin-4-yl)amino)-3,6,9,12-tetraoxatetradecyl)-2-ethoxypropanamide (128 mg, 44%) as a yellow solid. **^1^H NMR** (500 MHz, DMSO-*d*_*6*_) δ 1.02 (t, *J* = 7.0 Hz, 3H), 1.98–2.06 (m, 1H), 2.21 (s, 3H), 2.46–2.63 (m, 2H), 2.75–3.00 (m, 3H), 3.14–3.36 (m, 5H), 3.37–3.58 (m, 15H), 3.61 (t, *J* = 5.4 Hz, 2H), 3.88 (dd, *J* = 7.6, 4.7 Hz, 1H), 5.06 (dd, *J* = 12.7, 5.4 Hz, 1H), 5.16 (s, 2H), 6.60 (t, *J* = 5.7 Hz, 1H), 7.03 (d, *J* = 7.0 Hz, 1H), 7.12 (d, *J* = 8.6 Hz, 1H), 7.22 (d, *J* = 7.6 Hz, 1H), 7.28 (t, *J* = 7.6 Hz, 1H), 7.35 (d, *J* = 8.3 Hz, 2H), 7.44–7.51 (m, 2H), 7.53–7.60 (m, 3H), 7.72 (t, *J* = 5.8 Hz, 1H), 11.10 (s, 1H); **^13^C NMR** (125 MHz, DMSO-d_6_) δ 12.6, 15.0, 22.2, 31.0, 38.1, 38.4, 41.7, 48.6, 65.1, 68.8, 68.9, 69.5, 69.7, 69.77, 69.79, 69.8, 74.4, 80.4, 109.3, 110.7, 117.4, 120.8, 123.9, 126.8, 128.1, 130.0, 130.3, 131.2, 132.1, 135.5, 136.2, 137.6, 138.0, 146.4, 154.9, 167.3, 168.9, 169.6, 170.1, 171.2, 172.8; **MS (ESI**) *m/z* calculated for [C_43_H_53_BrN_5_O_11_]^+^ [M+H]^+^ = 894.2919, found = 894.2; **HRMS (ESI**) *m/z* calculated for [C_43_H_53_BrN_5_O_11_]^+^ [M+H]^+^ = 894.2919, found = 894.2932.

#### (i) Chemistry general experimental

Reagents and solvents (all anhydrous HPLC-grade) were obtained from commercial suppliers and used without any further purification unless otherwise stated. All reagents were weighed and handled in air unless otherwise stated. Concentration under reduced pressure refers to the use of a rotary evaporator.

#### (ii) Spectroscopy

^1^H and ^13^C NMR spectra were recorded using a Bruker Avance Neo spectrometer at a proton frequency of 500 MHz. All ^1^H and ^13^C NMR are quoted in ppm for measurement against TMS or residual solvent peaks as internal standards. Unless otherwise stated, all experiments were carried out using DMSO-d_6_ as solvent. ^1^H NMR chemical shifts (*δ*) are given in ppm ± 0.01, and coupling constants (*J*) are given in Hz ± 0.1 Hz. The ^1^H NMR spectra are reported as follows: δ/ppm (multiplicity, coupling constant(s) *J*/Hz, number of protons). Multiplicity is abbreviated as follows: s = singlet, br s = broad singlet, d = doublet, br d = broad doublet, dd = doublet of doublets, t = triplet, dt = doublet of triplets, q = quartet, dq = doublet of quartets, quint = quintet, m = multiplet. ^13^C NMR chemical shifts (*δ*) are given in ppm ± 0.1.

#### (iii) Mass spectrometry

LC-MS experiments were performed using a Shimadzu LCMS-2020 with electrospray ionization in positive ion detection mode with 20ADXR pump, SIL−20ACXR autosampler, CTO−20AC column oven, M20A PDA Detector and LCMS 2020 MS detector. LC was run in two setups: (i) Halo C18 column (2.0 µm, 3.0 × 30 mm) in combination with a gradient (5–100% B in 1.2 min) of water and FA (0.1%) (A) and CH_3_CN and FA (0.1%) (B) at a flow rate of 1.5 ml min^−1^; (ii) Poroshell HPH C18 column (2.7 µm, 3.0 × 50 mm) in combination with a gradient (5–95% B in 2 min) of aqueous 46 mM ammonium carbonate/ammonia buffer at pH 10 (A) and MeCN (B) at a flow rate of 1.2 mL/min; (iii) Halo C18 column (2.0 µm, 3.0 × 30 mm) in combination with a gradient (5–95% B in 2 min) of water and TFA (0.05%) (A) and CH_3_CN and TFA (0.05%) at a flow rate of 1.5 ml min^−1^ (B). The Column Oven (CTO−20AC) temperature was 40.0°C. The injection volume was 1 µl. PDA (SPD-M20A) detection was in the range 190−400 nm. The MS detector was configured with electrospray ionization as ionizable source; acquisition mode: scan; nebulizing gas flow: 1.5 l min^−1^; drying gas flow: 15 l min^−1^; detector voltage: tuning voltage ± 0.2 kV; DL temperature: 250°C; heat block temperature: 250°C; scan range: 90.00–900.00 *m*/*z*.

#### (iv) High-resolution mass spectrometry

Accurate mass data of samples were obtained using HRMS system with Waters Acquity I Class UPLC and Xevo G2-XS Q-TOF (Waters Corp., Milford, MA, USA). The samples were separated on reversed phase ACQUITY UPLC BEH C18 column (2.1 × 50 mm, 1.7 µm) using gradient elution with 0.1% FA in H_2_O as mobile phase A and 0.1% FA in MeCN as mobile phase B. Acquity PDA Detector was in the range 210–400 nm. The injection volume was 0.1 µl. Analytes were separated by a gradient method. The column temperature was set at 40°C and the flow rate at 0.4 ml min^−1^. Instrument control and accurate mass data were processed using Masslynx software. The MS data were acquired in positive ionization mode with the following conditions: MS equipped with ESI, sensitivity mode, capillary voltage at 2.5 kV, sampling cone voltage at 40.0 V, source temperature at 100°C, cone gas flow at 50 l h^−1^, desolvation gas flow at 600.0 (l h^−1^), acquisition mass range of 50–1200 Da.

#### (v) Preparative HPLC

Preparative HPLC was performed with a Waters MassLynx system with integrated MS detection and equipped with Prep C18 OBD 5 µm 30 × 150 mm columns from XBridge.

#### (vi) Compound naming

Compound names are those generated by ChemDraw 19.0.

### (c) Bioluminescence experiments

For longitudinal bioluminescence recordings in U2OS cell lines, cells were grown in black clear-bottomed 96-well plates (Greiner, 655090), covered with gas-permeable seals and containing 150 µl standard culture media, as described above. Cells were grown to confluence and maintained in 12 h:12 h 37°C:32°C cycles prior to the start of recording, at which point cells were synchronized with a full media change to standard media also containing 300 µM (1 mM for figures 1e, 2c and electronic supplementary material, figure S2*c*) d-luciferin (FL08608, BIOSYNTH). Bioluminescence recordings were all performed at 37°C and 5% CO_2_ using an ALLIGATOR system [46] (Cairn), with images being taken once every 30 min.

Bioluminescence recordings in PER2::LUC mouse lung fibroblasts in figure 2*e*,*f* were performed as above, but without gas exchange; at the start of recording, media was replaced with MOPS-buffered DMEM (high glucose, no phenol red; 31053-028, Gibco) supplemented with 20 mM MOPS, 1× GlutaMax^TM^ (35060-061), 10 mM sodium pyruvate (11360-070, Gibco), penicillin (100 units ml^−1^), streptomycin (100 µg ml^−1^; Gibco) and 10% HyClone FetalClone III serum (Cytiva), pH adjusted to 7.6 and osmolarity adjusted to 350 mOsm kg^−1^, with 1 mM d-luciferin added. Images were taken once every 20 min.

All recordings ran for approx. 24 h prior to the addition of experimental compounds, which were added at 10–20× concentration diluted in serum-free media (except in figure 1*e* in which treatments were added with the medium change). Medium changes and experimental manipulations were performed within 2.5 h of the time of transition from 37°C to 32°C in U2OS cells and *vice versa* for fibroblasts [53], during which cells were maintained at 37°C on a bespoke heating unit.

### (d) Bioluminescence data analysis

Bioluminescence quantification was performed using FIJI [91]; images were denoised and integrated density of individual wells (regions of interest) measured over time to generate luminescence values (reported in relative light units, RLU). Where necessary, background luminescence intensity was subtracted for each image, and individual traces were normalized to a sensible pre-treatment timepoint to correct for signal differences arising from differences in distance or angle with respect to the camera.

To estimate circadian parameters (including period, ‘*p*’), we fitted a damping cosine curve with the below equation by least-squares regression to each curve, manually inspecting the accuracy of the fit in each case.


$$y=(mx+c)+ae^{-kx}\text{cos}(2\Pi (x-r)/p)$$

### (e) Commercial compounds used in bioluminescence experiments

ARV−825 (cat. no.: 21109), dBET1 (18044) and MLN4924 (15217) were purchased from Cambridge Bioscience/Cayman Chemical; ARV−771 (HY−100972), AT1 (HY−111433) were purchased from Generon/MedChemExpress. For figure 2, pomalidomide (16534504) was purchased from Thermo Scientific.

### (f) Immunoblot experiments

For immunoblot assays, U2OS PER2::LUC (figure 1 and electronic supplementary material, figure S1) or WT cells (figure 2) were grown to confluence in 10 cm dishes in 7–8 ml medium prior to treatment and maintained at constant 37°C/5% CO_2_ throughout, apart from at treatment times. For figure 1*f* and electronic supplementary material, figure S1e experiments, treatments entailed a full media change to standard media buffered with 20 mM HEPES (reduced to 1% serum) and compounds at the noted concentrations. For figure 2*a* and electronic supplementary material, figure S1e, a complete media change was performed at the first treatment time, replacing with standard 10% serum media (described above) and the second treatment was supplementally added at 1000× in DMSO.

Cells were lysed on ice in RIPA lysis buffer containing 1 cOmplete™, EDTA-free protease inhibitor cocktail tablet per 10 ml buffer (04693159001, Roche). Lysates were centrifuged at 16000×*g* and supernatant was loaded onto 4–12% Bis Tris NuPAGE SDS-PAGE gels (Invitrogen) prior to transfer to nitrocellulose membranes. Membranes were blocked with 5% milk for 1 h, followed by overnight 4°C incubations with primary antibodies. Membranes were washed with TBST before and subsequent to 1 h incubations with HRP-conjugated secondary antibodies. Primary antibodies used (all at 1 in 1000): CRY1 was a kind gift from Katja Lamia, CRY2 (Bethyl Laboratories, A302-615A), β-actin (Santa Cruz, sc-47778). Secondary antibodies used (all at 1 in 10 000): anti-mouse IgG (Sigma, A4416) and anti-rabbit IgG (Sigma, A6154). Proteins were detected using 5 min incubations with Immobilon Western Chemiluminescent HRP Substrate (Millipore). Band quantification was performed in FIJI and ImageLab (Bio-Rad).

### (g) Cell viability assay

Assay was performed using a Cell Countess (Thermo Scientific). U2OS cells were grown to confluence in six-well plates (3.5 cm diameter). Media was replaced with 2 ml standard media containing respective **C8** concentrations for 72 h at constant 37°C. The monolayer was detached using Trypsin (0.25%)/EDTA (0.05 M), prior to being diluted to 60% in standard media. This solution was mixed 1 : 1 ratio with trypan blue followed by immediate recording of live and dead cell counts.

### (h) Quantitative mass spectrometry

#### (i) WT and CKO mouse fibroblasts experimental protocol

WT and CKO mouse fibroblasts (passage 16 and 14, respectively) [8] were grown to confluence in six-well plates (3.5 cm diameter) and maintained at 37°C throughout (apart from at treatment times) in standard media containing 10% serum (FBS; 10270106, Gibco). Cells of each genotype were then treated with either 1 μM **C8** or vehicle treatment (DMSO to 0.01%) as follows: at the start of the experiment (day 1) media was aspirated, cells washed with 1 ml phosphate buffered saline (PBS) and replaced with 1 ml media as above, supplemented with the compound or vehicle. Further identical media changes were performed on three additional days (3, 6 and 9). All media changes took place at the same time (within 1 h) on each respective treatment day. On day 10, cells were lysed and total protein extracted. We generated four replicate samples for each condition (16 in total), with each replicate comprising protein extracted from cell monolayers from two independent wells of a six-well plate.

#### (ii) Cell lysis and protein extraction

Cells were lysed at room temperature in 8 M urea buffer (8 M urea in 20 mM Tris, pH8) containing 1 cOmplete™, EDTA-free protease inhibitor cocktail tablet per 10 ml buffer (04693159001, Roche); media was aspirated, cells were washed with 2 ml PBS and then incubated in 100 μl urea buffer for at least 15 min. Cells were then scraped, collected, snap frozen in liquid nitrogen and stored at −80°C.

Lysates were later thawed on ice, given four rounds of 30 s sonication (Diagenode Bioruptor® Plus), and centrifuged for 15 min at 4°C at 16 000×*g* and supernatant collected. Extracted protein was then quantified using a Pierce™ BCA Protein Assay Kit (Thermo Scientific, 23225) following the manufacturer’s protocol. Samples were stored at −80°C.

#### (iii) Sample digestion, TMT-labelling and fractionation

Protein samples (50 µg) were diluted to 4 M urea and reduced with 5 mM DTT at 56°C for 30 min and alkylated with 10 mM iodoacetamide in the dark at room temperature for 30 min. Excess iodoacetamide was quenched by the addition of 5 mM DTT for 10 min. The samples were then diluted to 2 M urea and digested with Lys-C (Promega) at a ratio of 1 : 50 enzyme : protein, for 4 h at 25°C. Next, the samples were further diluted to 1.5 M urea and trypsin (Promega) at a ratio of 1 : 60 enzyme : protein, was added and incubated overnight at 30°C. Digestion was stopped by the addition of formic acid (FA) to a final concentration of 0.5%. Any particulate matter was removed by centrifugation at 16 000×*g* for 5 min. Supernatants were desalted using homemade C18 stage tips (3 M Empore) filled with 1.2 mg of Oligo R3 (Thermo Scientific) resin. Stage tips were equilibrated with 80% acetonitrile (MeCN)/0.5% FA followed by 0.5% FA. Bound peptides were eluted with 30–80% MeCN/0.5% FA and lyophilized.

Dried peptide mixtures from each condition were resuspended in 30 µl of 200 mM Hepes, pH 8.5. Isobaric labelling of the peptides was performed using TMTpro 18-plex reagents (Thermo Fisher Scientific); 15 µl reagent, reconstituted in anhydrous acetonitrile, was added and samples incubated at room temperature for 1 h. The labelling reaction was then quenched by incubation with 5% hydroxylamine for 30 min. TMT-labelled peptides were pooled into a single sample and desalted using stage tips as described above.

High-pH fractionation of labelled peptides was performed by off-line, high-pressure liquid chromatography (HPLC) using an XBridge BEH130 C18, 5 µm, 2.1 × 150 mm (Waters) column with XBridge BEH C18 5 µm Van Guard Cartridge, connected to an Ultimate 3000 Nano/Capillary LC System (Dionex). Peptides were separated with a gradient of 1–90% B in A (A: 5% MeCN/10 mM ammonium bicarbonate, pH 8; B: MeCN/10 mM ammonium bicarbonate, pH 8, [9:1]) in 1 h at a flow rate of 250 µl min^−1^. Eluted peptides were collected at 1 min/fraction, 54 fractions were collected and concatenated into 18 fractions and lyophilized. Dried peptides were resuspended in 1% MeCN/0.5% FA, desalted using C18 stage tips and partially dried down by vacuum centrifugation.

#### (iv) Mass spectrometry analysis

The fractionated peptides were analysed by LC-MS/MS using a fully automated Ultimate 3000 RSLC nano System (Thermo Fisher Scientific) fitted with a 100 μm × 2 cm PepMap100 C18 nano trap column and a 75 μm × 25 cm, nanoEase *m*/*z* HSS C18 T3 column (Waters). Peptides were separated using a binary gradient consisting of buffer A (2% MeCN, 0.1% FA) and buffer B (80% MeCN, 0.1% FA), at a flow rate of 300 nl min^−1^. Eluted peptides were introduced directly via a nanoFlex ion source into an Orbitrap Eclipse mass spectrometer (Thermo Fisher Scientific). The mass spectrometer was operated in real-time database search (RTS) with synchronous-precursor selection (SPS)-MS3 analysis for reporter ion quantification.

MS1 spectra were acquired using the following settings: resolution = 120 K; mass range = 400–1400 *m*/*z*; AGC target = 4e5; MaxIT = 50 ms and dynamic exclusion was set at 60 s. MS2 analysis was carried out with HCD activation, ion trap detection, AGC = 1e4; MaxIT = 50 ms; NCE = 33% and isolation window = 0.7 *m*/*z*. RTS of MS2 spectrum was set up to search the UniProt *Mus musculus* proteome, with fixed modifications cysteine carbamidomethylation and TMTpro 16-plex at N-terminal and Lys residues. Met-oxidation was set as variable modification. Missed cleavage = 1 and maximum variable modifications = 2. In MS3 scans, the selected precursors were fragmented by HCD and analysed using the Orbitrap with settings as follows: isolation window = 1.3 *m*/*z*; NCE = 55, orbitrap resolution = 50 K; scan range = 110–500 *m*/*z*; MaxIT = 200 ms and AGC = 1 × 10^5^. The acquired raw files from LC-MS/MS were processed using MaxQuant [92] with the integrated Andromeda search engine (v.1.6.17.0). MS/MS spectra were quantified with reporter ion MS3 from TMTpro 18-plex experiments and searched against the UniProt *Mus musculus* proteome fasta database (downloaded on November 2020). Carbamidomethylation of cysteines was set as fixed modification, while methionine oxidation and protein N-terminal acetylation were set as dynamic modifications. Tryptic digestion of up to two missed cleavages was allowed.

#### (v) Mass spectrometry data analysis

The MaxQuant output file was first processed with Perseus software (v 1.6.15.0); data were filtered to remove identifications from the reverse database, identifications with modified peptides only, and common contaminants. Data were exported to Microsoft Excel, and proteins with zero values in all samples were removed. Protein abundance was then normalized to a scaling factor such that the sums of each TMT channel were equivalent (scaling to the sum of the lowest channel). Data were filtered to include only proteins with abundance > 0 in at least 1 WT vehicle-treated sample (resulting in 7559 proteins).

Absolute fold change was calculated from these values, taking the average abundance for each protein in vehicle-treated CKO and wild-type vehicle-treated conditions and dividing the larger by the smaller. For abundance comparisons, data were transformed (log_2_ (abundance value+1)) and significant differences were calculated using *t*-tests (with Benjamini–Hochberg-corrected *q*-value < 0.05 used as a significance threshold). For Gene Ontology (GO) [64,65] analysis, enriched terms were identified using GOrilla [66,67] to compare unranked lists of proteins for which abundance significantly differed from the described vehicle treatment with a background list of all 7559 proteins. To reduce redundant terms in figure 3, and electronic supplementary material, figure S3, we also used ReviGO [93] and further manual curation.

#### (vi) U2OS cells mass spectrometry experiment

Cells were grown to confluence and maintained at 37°C throughout (apart from at treatment times) in standard media containing 10% HyClone FetalClone II serum (Cytiva). Each replicate comprised 1 × 10 cm dish of U2OS cells. At the start of the experiment, media was replaced and cells were incubated in 10 ml media containing either 1 μM **C8** or a vehicle treatment (0.01% DMSO) for 24 h prior to lysis.

Lysis and storage was performed as in fibroblasts, using 500 μl 8 M urea buffer. Storage, further processing, mass spectrometry and initial analysis were performed as in fibroblasts, except that the UniProt human reviewed fasta database (downloaded March 2019) was used for database searches.

Data from Perseus software (v 1.6.15.0) were exported to Microsoft Excel, and any proteins with zero values in all replicates in either condition were removed. Protein abundance was then normalized to a scaling factor such that the sums of each TMT channel were equivalent (scaling to the sum of the lowest channel). Absolute fold change was calculated from these values, taking the average abundance for each protein in **C8** and vehicle-treated conditions and dividing the larger by the smaller.

For statistical comparisons, abundance values were transformed (log_2_ (abundance value+1)) and significant differences were determined by two-way ANOVA, comparing protein ID and treatment in each replicate condition and performing multiple comparison tests using the two-stage step-up method of Benjamini Krieger and Yekutieli. Enriched GO [64,65] function terms were identified using the ranked ontology method in GOrilla [66,67], including only significantly upregulated proteins and filtering for those with a passing threshold of >10% increase in abundance.

#### (vii) General statistics, analysis and figures

Unless noted, data handling and statistics were performed using Microsoft Excel (Microsoft Corporation) and Prism GraphPad 9/10 (Boston, MA, USA). Bioluminescence data were analysed using FIJI [91], Microsoft Excel and Prism GraphPad. Prism Graphpad and Adobe Illustrator (Adobe Inc.) were used to generate most data figures. Figure 3*b* was generated using ggplot2 [94] in RStudio [95,96]. Figure 1 schematics were generated using BioRender.com.

## Data Availability

Data is provided as electronic supplementary material [97]. The mass spectrometry proteomics data have been deposited to the ProteomeXchange Consortium via the PRIDE partner repository with the dataset identifier PXD058309.
